# Co-doped In-Situ Engineered Carbon Nano-Onions Enabled High-Performance Supercapacitors

**DOI:** 10.3390/nano13010019

**Published:** 2022-12-21

**Authors:** Debananda Mohapatra, Mostafa Saad Sayed, Jae-Jin Shim

**Affiliations:** 1 School of Materials Science and Engineering, Yeungnam University, Gyeongsan 38541, Republic of Korea; 2 School of Chemical Engineering, Yeungnam University, Gyeongsan 38541, Republic of Korea

**Keywords:** in-situ doping, graphitization, carbon nano-onions, fullerene, supercapacitors

## Abstract

The feasibility of achieving in situ sulfur (S) and nitrogen (N) co-doped carbon nano-onions (CNOs and SN–CNOs) via a simple flame-pyrolysis technique without using sophisticated high-vacuum annealing or expensive nanodiamond-based complex processes is demonstrated for the first time. The characteristic onion-like feature of 0.34 nm remained intact with a high degree of ordering and graphitization, even though the S and N heteroatoms were co-doped simultaneously. The in situ co-doped SN–CNO demonstrated high supercapacitor device performance with a high energy density of 25 Wh kg^−1^ at a maximum power density of 18 kW kg^−1^, maintaining 98% specific capacitance over 10,000 cycles at 10 A g^−1^. These are the highest achieved device performance values of a fullerene family electrode material to date.

## 1. Introduction

Carbon-based nanostructures are outstanding supercapacitor electrode materials, especially for electric double-layer capacitors, owing to their high specific power, long cycle life, and, most importantly, low maintenance cost. Various carbon nanomaterials, such as graphene, carbon nanotubes (CNTs), carbon nano-onions (CNOs), heteroatom-modified graphene, and nanofibers, have been investigated for the same use, considering their outstanding electrochemical properties [1,2]. The electrical and electrochemical properties of graphene and CNTs have been altered and improved significantly via heteroatom doping and co-doping with N, S, B, and P. Many studies have been performed and reported in the recent past [2,3,4]. Specifically, in the case of CNO, which is a multilayered graphitic shell-shaped material belonging to the fullerene family, this is performed under post-synthesis conditions with sophisticated instrumentation. CNOs were first produced using expensive nanodiamond precursors requiring high-energy synthesis protocols, such as detonation and vacuum annealing (1800 °C, 10^−8^ Torr). Few studies have reported CNO doping with B, N, S, and P [5,6]. However, all of these studies used expensive nanodiamonds as a precursor for pristine CNOs. They later used multistage harsh post-doping chemical treatments, eventually damaging the CNO graphitic structure, thereby deteriorating the intrinsic electrical conductivity. To resolve this inherent limitation, we recently developed novel in situ N- and S-doped CNOs using neither an expensive precursor nor a post-synthesis multistage doping process [7,8]. Notably, the precursor materials used for N–CNOs or S–CNOs are either only acetonitrile (acts as both a carbon framework source and nitrogen dopant) [7] or thiophene (acts as carbon and sulfur dopant source) [8], which are inexpensive compared to nanodiamonds. These were the first reports on the feasibility of the in situ doping of CNO. Moreover, our adopted synthesis was conducted at room temperature and atmospheric pressure with no harsh chemicals or further purification steps, resulting in a lower input energy and capital cost. By contrast, in previous studies, even pristine CNO synthesis involved highly sophisticated instrumental conditions such as high-vacuum annealing (1800 °C at 10^−6^ Torr pressure). In [9], the heteroatom doping involved even further efforts with multistage post-doping chemical treatments [5,6].

More importantly, compared to other studies that used an expensive nanodiamond powder as a precursor and complex chemical oxidation and annealing at high temperatures (i.e., detonation technique), the current approach to synthesizing SN–CNO particles is facile, fast, and cost-effective. However, to the best of our literature search and background research, the in-situ synthesis of S and N co-doped CNOs and their application in supercapacitors have not been studied thus far. Therefore, we demonstrated their use in a symmetrical cell configuration.

## 2. Experimental

### 2.1. Materials

Acetonitrile (99%), thiophene (99%), tetraethylammonium tetrafluoroborate (TEABF_4_) salt, isopropanol (99.5%), and glass microfiber filters (Whatman, Grade GF/D) were purchased from Sigma–Aldrich and used without any further purifications.

### 2.2. Water-dispersible Sulfur and Nitrogen Co-doped Carbon Nano-onions (SN–CNOs) Preparation Method

**Figure 1:**
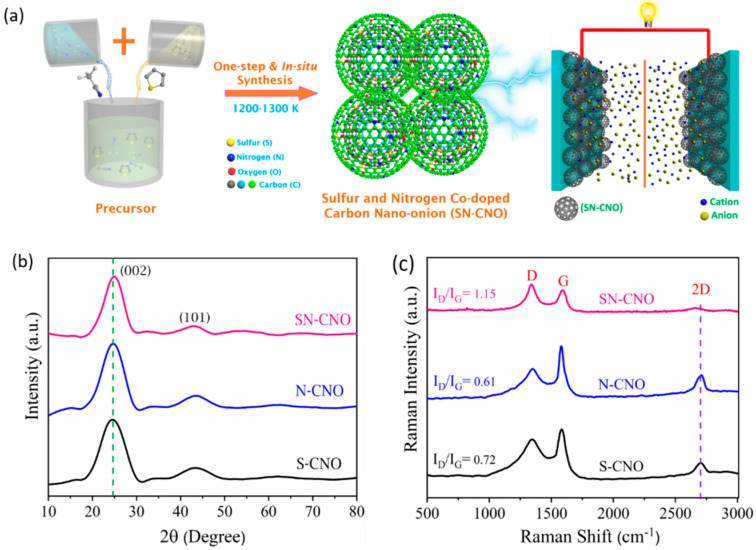
Schematic depiction of in situ sulfur and nitrogen co-doped carbon nano-onions (SN–CNOs), their structural ordering, and supercapacitor application: (**a**) simple, one-step synthesis scheme for SN–CNOs; (**b**) XRD and (**c**) Raman spectra of the as-synthesized S–CNOs, N–CNOs, and SN–CNOs.

A simple flame-pyrolysis method was employed to prepare the SN-CNO particles. Approximately 50 mL each of acetonitrile and thiophene was added to a small beaker and then mixed and placed, as per Figure 1a, and then burned in the air under the controlled conditions of a fume hood to avoid the release of toxic gases during synthesis. The flame temperature of this process in the air was in the range of 800–1000 °C [7,8]. In this study, acetonitrile and thiophene were employed as the sources of the dopant N, S, and the C backbone. The sample was deposited over a conical flask containing water for cooling, and a constant temperature was maintained, as discussed elsewhere [8]. Similarly, S-CNOs and N-CNOs were prepared following the methods reported elsewhere [7,8].

The as-prepared black carbon nanoparticles were collected. Later, they were utilized for material characterization and targeted applications without harsh post-processing chemical treatments or further purification steps. The flame pyrolysis experiment was performed under strict conditions, such as keeping a constant organic precursor concentration, temperature, separation between the substrate and flame, and collection period. The sample preparation time depends on the amount of precursor. This process is simple and leads to the repetitive formation of S and N heteroatom-doped carbon nanostructures with similar microstructural features and physical properties, resulting in the ability of the as-obtained nanomaterials to perform in various applications. This process can also be scaled up to the gram scale for other functional applications (e.g., metal-free carbocatalysts for water remediation, HER, and OER). A more detailed protocol is discussed in our current application for a patent on this material process (quality and quantity), the engineering, and the various functional applications. Therefore, from an intellectual property perspective, we did not discuss the full manuscript in detail.

### 2.3. As-Synthesized SN-CNOs’ Physiochemical Characterizations and Modeling Parameters

Field-emission transmission electron microscopy (FE-TEM, FEI Tecani G2 F20, The Netherlands) was used to explore the microstructure of the as-synthesized SN-CNOs. The surface morphology and the elemental composition of the as-synthesized SN-CNOs were further examined by a field emission scanning electron microscope equipped with an energy-dispersive X-ray analyzer (FE-SEM/EDX, Hitachi, S-4800, Tokyo, Japan). The X-ray photoelectron spectroscopy (XPS, Thermo Scientific, Boston, MA, USA) of the SN-CNOs was performed using Al K_α_ monochromatic radiation to investigate the surface chemical composition. An XploRA plus a HORIBA spectrometer, France equipped with 532 nm of laser excitation was employed to understand the vibration spectra of the SN-CNOs particles. The as-obtained SN-CNOs particles’ crystallinity was examined using X-ray diffraction (XRD, PANalytical, X’Pert-PROMPD, USA) with Cu K_α_ radiation. The SN-CNO particles’ three-dimensional atomistic design and modeling work was performed using Autodesk 3ds Max and Avogadro version 1.2.0 software. 

### 2.4. SN-CNO||SN-CNO Device Fabrication, Electrochemical Characterization, and Performance Evaluation

Electrochemical tests of the SN-CNO||SN-CNO device were conducted through two-electrode split cell configurations in 1M TEABF_4_ with acetonitrile. The electrode materials were prepared on carbon paper (CC, 1.0 × 4.0 cm, MTI Korea) and cleaned by immersing and sonicating in 3 M HCl, DI water, and ethanol before drying in a vacuum oven overnight at 60 °C. A homogeneous suspension of as-synthesized SN-CNOs was prepared in isopropanol without a binder element. A drop-casted method was followed to coat the precleaned CC. At a later stage, the dop-casted CC was dried overnight in a vacuum condition. Glass microfiber filter paper (Whatman, Grade GF/D) was placed between the active electrodes as a separator.

An Autolab (PGSTAT 302 N potentiostat/galvanostat, Utrecht, The Netherlands) workstation was applied to explore the electrochemical performance of the cell/device. The cyclic voltammetry (CV), galvanostatic charge–discharge (GCD), and electrochemical impedance spectroscopy (EIS) measurements were conducted at the open circuit potential in the frequency range of 0.01–100 kHz. The long cycling stability was investigated using a WBCS300L Battery Cycle System (WonAtech, Seoul, South Korea). All electrochemical measurements were performed at room temperature.

The specific capacitance (*C* in F g^−1^) of the SN-CNO||SN-CNO device was evaluated using the following Equation [8,9]:

(1) $$C=\frac{2I\times \int_{V_{i}}^{V_{f}}Vdt}{m\times V}$$

where *I* represent the discharge current (A); ***∫****V.dt* is the area under the discharge curve; *m* represents the total mass of the active material (g); *V_f_* and *V_i_* are the final and initial potential (*V*), respectively; and Δ*t* represents the discharge time (s).

The energy density (*E*, Wh kg^−1^) and power density (*P*, W kg^−1^) of the SN-CNO||SN-CNO device were evaluated as follows [9]:

(2) $$E=\frac{I\times \int_{V_{i}}^{V_{f}}Vdt}{m\times 3.6}$$

(3) $$P=\frac{\;E\times 3600}{\Delta t}$$

## 3. Results and Discussion

### 3.1. SN-CNO Microstructure and Surface Chemical Compositions

**Figure 2:**
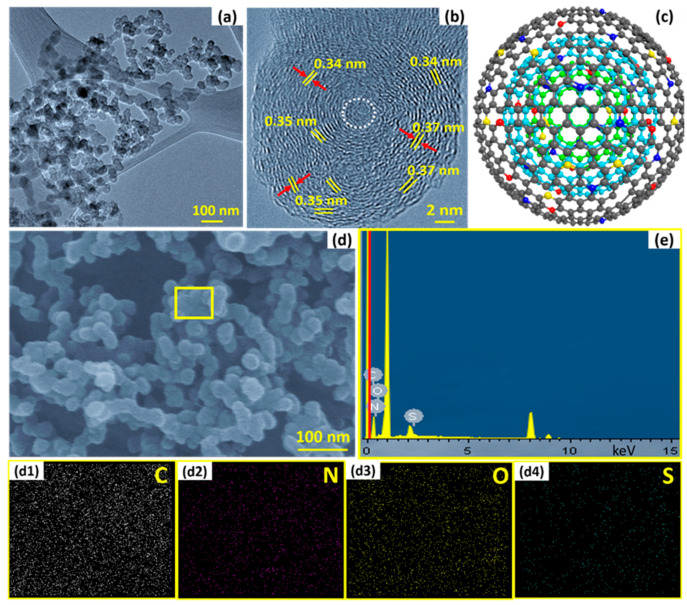
The as-synthesized SN–CNO particles’ graphitic nanostructure: (**a**) TEM images of as-synthesized SN-CNOs particles showing the interconnected network structure; (**b**) HR-TEM images of SN–CNO particles with unresolved cores (white, dotted circles) and well-maintained intergraphitic layers; (**c**) a single SN–CNO particle’s representative atomistic model (atoms are shown with the same colors as illustrated in Figure 1a); (**d**,**e**) FE-SEM overview and SEM–EDX spectroscopy of different elements (C, N, O, and S) of as-prepared SN–CNO particles and their corresponding elemental maps (**d1**–**d4**), respectively.

We briefly describe the SN–CNO synthesis protocol shown in Figure 1a. Incorporating the dual hetero-dopants into the CNO structure could influence their intrinsic graphitization and crystal structure. The graphitic structure and quality of the SN–CNO sample was investigated using XRD (Figure 1b) and later by Raman spectroscopy (Figure 1c). Two significant peaks were observed at approximately 25° and 44° for all of the samples, with little reduction in the peak height, shifting, and broadening. For the S–CNOs and N– CNOs, the prominent graphitic peak (002) due to the sp^2^ hybridized carbon was located at 2θ, 24.6°, corresponding to an intergraphitic layer distance of 0.34 nm; however, for the SN–CNO, it was slightly blue-shifted with an interlayer spacing of 0.37 nm. The SN–CNO major peak not only shifted to a lower angle but also became broad and more depressed by intensity, indicating the role of simultaneous N and S doping in the CNO structure. This behavior could also be interpreted as the result of more intrinsic strain induced in the CNO layers, causing some graphitic defects among those concentric layers, which are indicated in the HR-TEM image (Figure 2) and Raman spectra (Figure 1c). An interlayer spacing greater than 0.34 nm indicates the existence of defects in the graphitic structure. In line with this, we can clearly show that the broadness of the significant (002) graphitic peak supports the random distribution of the graphite layers in the SN–CNOs, with a short domain order. The higher interlayer spacing in the as-prepared SN–CNOs was due to the presence of structural defects. Conversely, there were no distinguishable peaks of any impurity phases or secondary phases, proving the in-situ co-doping, with some intergraphitic structural defects. The presence of defects in the SN–CNO graphitic layers, with exposed edges and curvature, could contribute to the improved electrochemical performance.

Figure 1c shows the comparative Raman spectra of the S–CNO, N–CNO, and SN–CNO as-prepared samples to clearly distinguish the shifting and broadening of the characteristic D-, G-, and 2D-bands. The presence of a defined D-band at 1340 cm^−1^ was induced by the local graphitic basal plane derivatization, which created an sp^3^ distortion. This can also originate from stacking disorders and graphitic edge defects caused during the formation of the CNO structure. As the sharp D-band (A_1g_) vibrational phonon modes in all of the samples corroborate, the defective nature of the CNOs may arise from the stacking faults and local curvature. This became more dominant and broader for the SN–CNO sample when compared to the N–CNO and S–CNO samples, which show more structural disorderliness. The difference between the samples can be clearly observed in the HR-TEM images (Figure 2) concerning the variations in the characteristic intergraphitic planar spacing. The G-band (E_2g_) originated at 1581 cm^−1^ from the sp^2^ hybridized graphitic carbon in the as-synthesized samples. The appearance is broader in the SN–CNO sample compared to the others, reflecting the influence of the co-doping in place of single N or S-doping. Not only did the G-band broaden but the D- and G-band also redshifted upon close observation. The broadening and red-shifting of the characteristic band spectra are essential indicators of the successful doping of both N and S into graphene-like CNO structures. The carbonaceous materials’ structural indicator I_D_/I_G_ band intensity ratio also increased from S-CNO (0.72) and N-CNO (0.61) to SN–CNO (1.15). The higher I_D_/I_G_ ratio of the SN–CNO sample is also an indicator of the presence or generation of vacancies/defects while both S and N were simultaneously doped into the CNO structure. Most importantly, the loss of the intensity and wideness of the 2D-band compared to the N–CNO and S–CNO (sharp and defined at 2705 cm^−1^) validates the introduction of more defects and domain randomization in the graphitic structure of the SN–CNO. Therefore, it is difficult to observe the high degree of crystal lattice perfection in the HR-TEM in terms of the concentric graphitic carbon shells compared to our previous reports [7,8].

The overall morphology, elemental composition, and internal microstructure of the as-prepared SN–CNO particles are shown in Figure 2, using high-resolution transmission electron microscopy (HR-TEM) and field-emission scanning electron microscopy (FE-SEM) equipped with energy-dispersive spectroscopy (EDS). The obtained particles were nearly spherical in shape and with a diameter size of approximately 35 ± 5 nm, interconnected with tight interparticle contacts (Figure 2a). These intimate tight interparticle contacts facilitate the ease of electron transport throughout the sample at their connecting boundary regions. The representative HR-TEM image (Figure 2b) reveals the concentric, interpenetrating graphitic layers and good intergraphitic separation from each other at a varying distance of 0.34–0.37 nm. As per our previous report [10], the characteristic feature of CNOs is their well-arranged concentric graphitic layer spacing of 0.34 nm, resembling planar bulk graphite. Similar features were also observed in in situ nitrogen [7] and sulfur-doped CNOs [8] without affecting the characteristic CNO feature and their intergraphitic spacing of 0.34 nm. Interestingly, in these simultaneously in situ nitrogen and sulfur co-doped CNOs, the features were slightly different from those of pristine CNOs, N-CNOs, and S-CNOs. The critical and distinctive features, such as the intergraphitic planar spacing, were modulated and varied between 0.33 and 0.37 nm owing to the co-doping effect, which was not observed before. The change in the spacing directly affected the intrinsic strain among the stacked graphitic layers with the introduction of the graphitic defects, as shown in the XRD and Raman sections. The yellow, dotted lines represent the probable nucleation and growth of the particle toward the outer curvature with graphene-like facets. Notably, the onion-like features were maintained even though the CNOs were co-doped. The presence of the sulfur and nitrogen dopants, along with the significant carbon and oxygen content, can be seen in the SEM–EDX spectral lines (Figure 2e) from the FE-SEM overview (Figure 2d) and their corresponding elemental mappings in Figure 2d1–d4.

**Figure 3:**
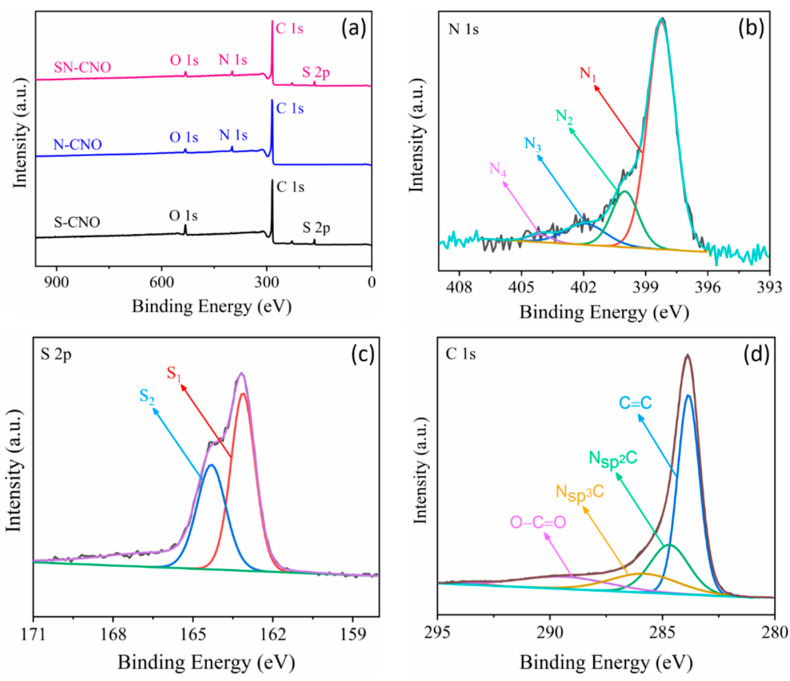
Surface composition of the S–CNOs, N–CNOs, and SN–CNOs: (**a**) comparative XPS survey spectra of the S–CNOs, N–CNOs, and SN–CNOs; high-resolution core XPS spectra of (**b**) N 1s, (**c**) S 2p, and (**d**) C1s.

The presence of these elements was also investigated and confirmed using X-ray photoelectron spectroscopy (XPS), as shown in Figure 3. The full XPS survey spectra (Figure 3a) clearly establish the presence of both the sulfur (165 eV) and nitrogen (399 eV) contents along with the major carbon (285 eV) and oxygen (532 eV), as compared to the previously reported in situ N–CNO [7] and S–CNO [8]. All of the other nitrogen and sulfur-doped CNO samples showed only dopant nitrogen (approx. 4 at.%) and sulfur (approx. 3.5 at.%) contents. By contrast, the SN–CNO sample undoubtedly indicates both sulfur (approx. 3.4 at.%) and nitrogen (approx. 5.1 at.%) content, as shown in Figure 3a. It can be confirmed, after observing the EDS spectra of the elemental distribution and the XPS survey spectra that both nitrogen and sulfur were incorporated into the CNO-layered structure satisfactorily. The presence of both the hetero-dopants effectively enlarged the characteristic intergraphitic layer distance from 0.34 (in the case of pristine CNOs, S–CNOs, and N–CNOs) to 0.37 nm. The larger interatomic radius of both of the dopants compared to the host carbon atom may lead to some graphitic defects in the SN–CNO structure. This could necessarily result in providing more electroactive sites for the electrolyte ions during the charge storage. The chemical nature of both nitrogen and sulfur, along with their heteroatomic bonding configuration, can be retrieved by deconvoluting the core N 1s (Figure 3b), S 2p (Figure 3c), and C 1s (Figure 3d) spectra. The core high-resolution N 1s spectra can be resolved into four different peaks that appear at 397.8, 400.1, 401.8, and 404.2 eV, representing the different chemical states of the dopant. These peaks could be attributed to the pyridinic (N_1_)-, pyrrolic (N_2_)-, graphitic (N_3_)-type, and nitrogen oxides or π-excitations (N_4_), respectively. N_1_- or N_2_-type nitrogen mostly contributes to the sp^2^-character of the graphene-like CNO structure that arises owing to the donation of π-electrons to the carbon network. These N_1_/N_2_-type nitrogens appeared to be located at the edge of the exposed graphene sheets or sometimes in the defect sites present in the CNO basal plane and were bonded to two adjacent carbon atoms. Conversely, the graphitic N_3_-type nitrogen was attributed to replacing central carbon atoms at both the CNO edge surface and graphene-like basal planes. The replacement of the central carbon and the valley/terminal carbon atoms led to the transfer of the N-dopant electrons to the π-system of the CNO framework, effectively increasing the SN–CNOs’ overall electronic conductivity. Other peaks occurred at higher binding energies, such as the N_4_-type nitrogen, possibly from the π–excitations or nitrogen oxides. In line with this, the high-resolution S 2p core spectra can be further deconvoluted into S ^2^p_3/2_ (S1) and S ^2^p_1/2_ (S2) doublets that appeared at 163.2. and 164.3 eV, respectively. This can be ascribed to the thiophenic structures attributed to C=S and C−S bonds with neighboring aromatic carbon atoms, respectively, without any higher binding energy C–SO_x_ states, confirming the S-doping of the CNO network by sulfur [8]. The results of both the N 1s and S 2p core XPS spectra essentially demonstrate that the doped sulfur and nitrogen were predominantly incorporated into the CNO framework of the as-synthesized SN–CNO samples without necessitating any post-purification and external doping steps. The high-resolution C 1s core spectra revealed that the major peak was centered at 284.4 eV, corresponding to the sp^2^ C hybridized carbon atoms in the SN–CNO sample. In contrast, the peaks at the higher binding energies of 285.3, 285.9, and 289 eV indicated the presence of N–sp^2^ C, S–sp^2^ C, and residual C=O or O−C=O groups, respectively. The broad asymmetric nature of the higher binding energy peaks sustained the dual doping, maintaining a high concentration of sp^2^ character, which is crucial for increasing the SN–CNOs’ intrinsic electrical conductivity. In addition to the intimate intergraphitic contacts seen in Figure 2b, electron kinetics is a vital requirement of any carbonaceous material. Additionally, carbonyl and carboxylic functional groups in the as-prepared SN–CNO samples support high hydrophilicity. Therefore, harsh post-synthesis chemical treatment or prolonged activation can be eliminated, which are indispensable for nanodiamond-derived CNOs and their post-doping processes.

### 3.2. SN–CNO||SN–CNO Device Performance Studies

**Figure 4:**
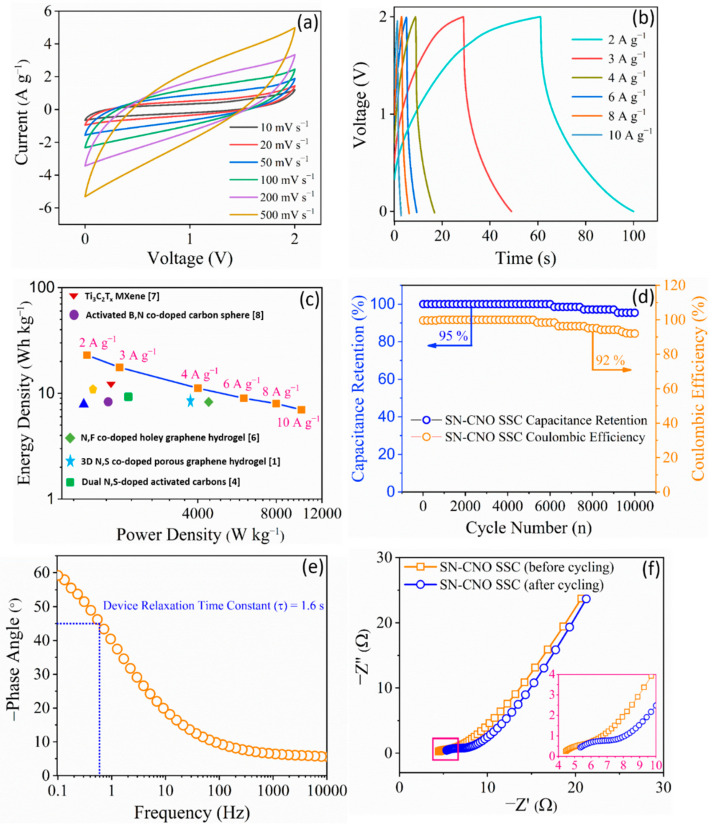
SN–CNO||SN–CNO symmetric supercapacitor (SSC) electrochemical device performance in 1 M TEABF_4_ with ACN: (**a**) cyclic voltammograms recorded at different scan rates from 10 to 500 mV s^−1^; (**b**) their corresponding GCD curves obtained at 2 to 10 A g^−1^ current densities; (**c**) Ragone plot relating the energy density and power density of the SSC in comparison to recent reports [10,11,12,13,14,15,16,17]; (**d**) long-term cycling stability and Coulombic efficiency up to 10,000 continuous charge–discharge cycles at 10 A g^−1^; (**e**) Bode plot via EIS showing the device response; (**f**) Nyquist plots via EIS recorded before and after 10,000 long-term continuous charge–discharge cycles of the device.

There was a tradeoff between the electrolyte voltage window and the ionic conductivity. Aqueous electrolyte-based supercapacitors suffer from a limited operation voltage window, hence the energy density. Therefore, the novel SN–CNO-based supercapacitor was directly tested in a two-electrode symmetric configuration in 1 M TEABF_4_ with ACN. The nonavailability of the redox behavior and the nearly rectangular nature of the obtained CV curves in Figure 4a show that the charge storage was mainly due to the electric double layers at the electrode/electrolyte interface. Different scan rates ranging from 10–500 mV s^−1^ at a voltage window of 2 V were recorded for the electrochemical performance evaluation. The slight deviation of the rectangular shape may be due to the presence of the dual N and S dopants in the carbon structure instead of oxygen-containing functionalities. This is because the percentage of both the individual N and S dopants is higher than that of the oxygen content. The observed CV curves are congruent with their counter GCD curves of 2–10 A g^−1^ (Figure 4b). The GCD curves are quite linear and symmetrical at higher current densities than those with a lower current density. The nanosized nature of SN–CNO (~35 nm) benefits the huge electroactive accessible surface area, facilitating an ample amount of charge storage at a given mass. The internal resistance of the bulk SN–CNO electrode sample will also substantially decrease because of the shorter diffusion path length supported by the nano dimension and curvature. However, the slanted CV at a very high scan rate may have appeared because of the electrolytic resistance offered by the organic electrolytes. In other words, those ions experienced a higher resistance offered by the pores at a shorter time due to the high scan rate; hence, the overall resistance of the resulting device increased. This led to a slight deviation from rectangularity.

Figure 4c shows a Ragone plot of the SN–CNO||SN–CNO in relation to its energy and power density. At 2 A g^−1^, it delivered an energy density of 25 Wh kg^−1^ at a power density of 1500 W kg^−1^. The device also maintained a high power density of 12,000 W kg^−1^ at an energy density of 10 Wh kg^−1^, even when drawing five times more current. These data sets are quite competitive with some recent reports on co-doped graphene [11], graphene aerogel [12], carbon spheres [13], porous carbon [14], activated carbons [15,16], MXene [17], etc. The long durability of the device is a crucial criterion for its practical use.

The SN–CNO||SN–CNO’s long-term cyclic performance is shown in Figure 4d, exhibiting an excellent cyclic stability of 95% with a 92% coulombic efficiency even after 10,000 cycles. Another essential requirement of a practical operational supercapacitor is that it should perform at a very high current load, ensuring peak power delivery. This is characterized by the supercapacitor figure of merit derived from the Bode plot (Figure 4e). A supercapacitor is expected to have a short relaxation time (τ_0_ = 1/f_0_) so that it yields a faster power discharge. As depicted in Figure 4e, SN–CNO||SN–CNO was found to have a relaxation time of 1.6 s. Electrochemical impedance spectroscopy (EIS) was used to estimate the resistive parameters of the device, which arise from the electrode and electrolyte and various resistance factors. The ESR of the SN–CNO||SN–CNO device was found to be 4.45 Ω, and it marginally increased to 5.35 Ω when it was checked again after 10,000 cycles, as shown in Figure 4f.

The device performance could be attributed to (i) the presence of both hetero-dopants (i.e., S and N), (ii) the high degree of curvature-induced graphitization, and (iii) the nano dimension (~35 nm) caused by increasing the surface-to-volume ratio and the shortening of the diffusion path. Both the N and S dopant atoms not only modified the electron distribution in the CNO structure but also increased the electrolyte ions’ surface wettability. This leads to increased access and participation of electroactive species, allowing more ion accommodation at the electrode surface. The heteroatom co-doping may have also augmented the number of capacitive sites and surface wettability due to the existence of both carboxylic and hydroxylic functionalities.

## 4. Conclusions

In summary, we successfully synthesized in situ co-doped CNOs in bulk quantity using a one-step and straightforward flame-pyrolysis method by eliminating the use of expensive precursor materials with the associated sophisticated equipment. We not only introduced in situ co-doped CNO synthesis but also demonstrated its potential application as a practical supercapacitor device. The SN–CNO||SN–CNO symmetric supercapacitor exhibited competitive performance compared to other well-known carbon allotropes, such as MXene. It also showed significant durability. Our findings also offer a new reference and novel approach for further developing economical metal-free fullerene carbon nanostructures. Our results confirm that in situ co-doped CNO could be a potential candidate for use in other carbon allotropes by opening a new avenue for developing clean energy storage technologies, especially for supercapacitors and electrocatalytic applications.
